# Diversity and Structure of Bacterial Communities in Different Rhizocompartments (Rhizoplane, Rhizosphere, and Bulk) at Flag Leaf Emergence in Four Winter Wheat Varieties

**DOI:** 10.1128/mra.00222-22

**Published:** 2022-04-13

**Authors:** Athanasios Zervas, Lea Ellegaard-Jensen, Rosanna C. Hennessy, Frederik Bak, Ying Guan, Courtney Horn Herms, Kitzia Yashvelt Molina Zamudio, Dorthe Thybo Ganzhorn, Dorette Sophie Müller-Stöver, Jabeen Ahmad, Amy Grunden, Carsten S. Jacobsen, Mette Haubjerg Nicolaisen

**Affiliations:** a Department of Environmental Science, Aarhus University, Aarhus, Denmark; b Department of Plant and Environmental Science, University of Copenhagen, Copenhagen, Denmark; c Department of Plant and Microbial Biology, North Carolina State University, Raleigh, North Carolina, USA; University of Maryland School of Medicine

## Abstract

Understanding basic interactions at the plant-soil interphase is critical if we are to exploit natural microbial communities for improved crop resilience. We report here 16S amplicon sequencing data from 3 rhizocompartments of 4 wheat cultivars grown under controlled greenhouse conditions. We observed that rhizocompartments and cultivar affect the community composition.

## ANNOUNCEMENT

Wheat (Triticum aestivum L.) is an essential food crop and globally ranks as the second most important grain crop used in the food, feed, and raw materials industries (http://www.fao.org/statistics/en/). Interactions in the rhizosphere may lead to improved plant performance and resistance to biotic and abiotic factors (1). The effects of different soils on the wheat root microbiome composition have recently been studied (2), but there is limited knowledge about the effects of wheat cultivars on microbial communities within different rhizocompartments. Here we report on the diversity and structure of the associated bacterial and archaeal communities based on amplicon sequencing of 16S rRNA gene libraries constructed from 3 rhizocompartments sampled from four wheat cultivars.

A plant growth experiment was conducted under controlled greenhouse conditions. Field soil was collected from the plough layer (0 to 25 cm) at the University of Copenhagen’s experimental farm in Taastrup, Denmark (details about soil characteristics are described elsewhere [3]). Polyvinyl chloride (PVC) pots (height, 24 cm; diameter, 7 cm) were prepared for planting by adding field soil premixed with sterile sand (DANSAND; filter sand no. 1) (2:1 ratio). Seeds of winter wheat cultivars Heerup, Sheriff, Kvium, and Rembrandt (Sejet Plant Breeding, Denmark) were sown in soil-filled pots with 105 individuals per cultivar (1 plant per pot). The different wheat cultivars were subsequently sampled at the flag leaf emergence stage (Feekes 8.0 [4]) at day 141 (Kvium), 142 (Sheriff), 145 (Rembrandt), or 146 (Heerup) after sowing. Plants were scored based on plant height as an indicator of plant performance, and the five tallest and shortest plants were sampled for each variety. For each plant, we collected two rhizocompartments (rhizosphere and rhizoplane) in addition to bulk soil samples. Samples were flash frozen, freeze-dried, and kept at 4°C until DNA extraction.

**FIG 1:**
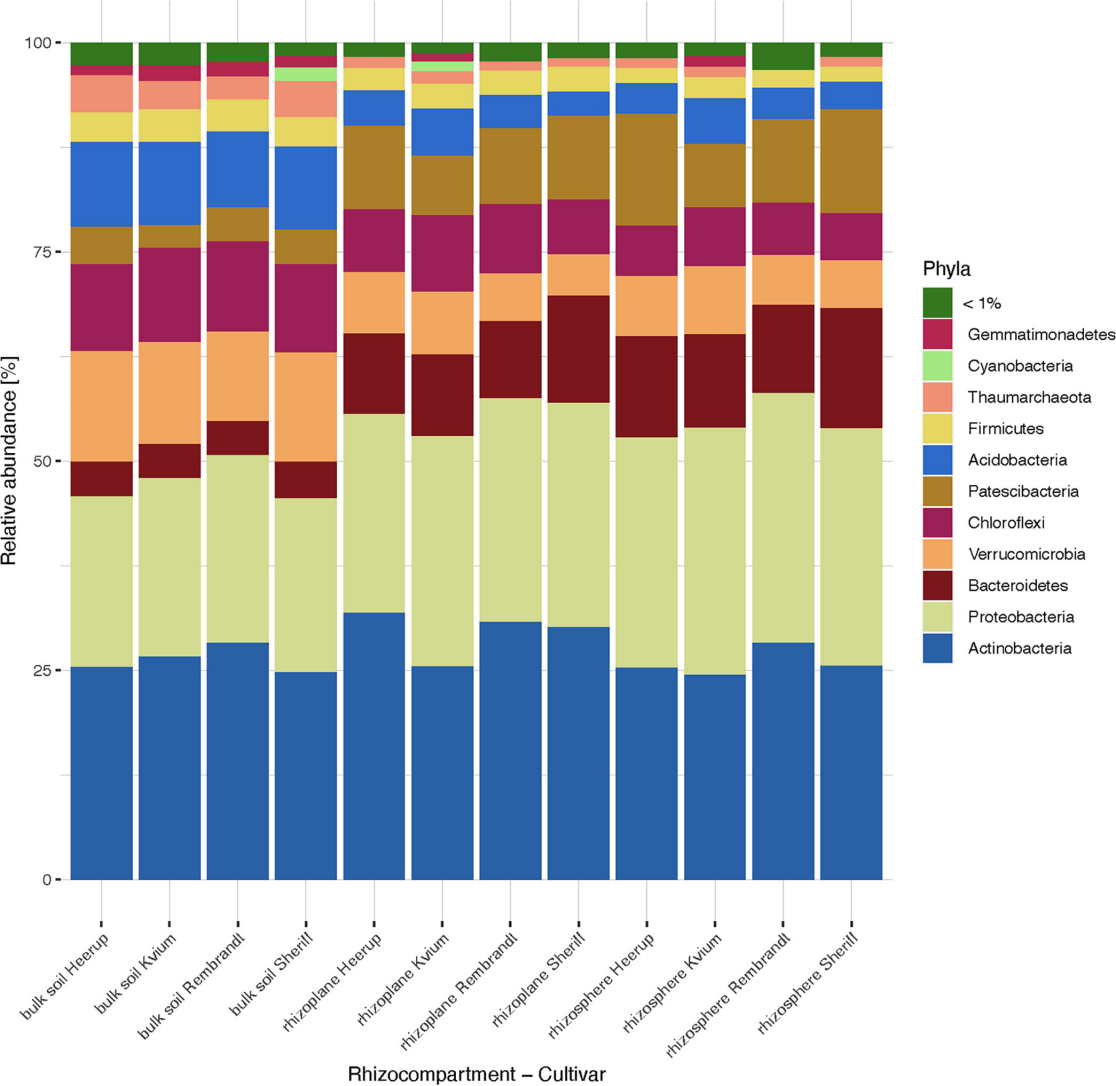
Microbial composition of the 3 rhizocompartments of the 4 wheat cultivars used in the study. The relative abundances of the main phyla (relative abundance > 1%) as identified by 16S amplicon sequencing are shown.

DNA was extracted using the DNA protocol from the NucleoBond RNA soil minikit (Macherey-Nagel, USA), and concentration was measured on a Qubit 4. Amplicon libraries for Illumina sequencing were prepared by a 2-step PCR (5) with the 341F-806R primer pair targeting the V3-V4 hypervariable regions of the 16S rRNA gene (6) using PCR Ultra mix (PCR Biosystems, UK). The samples were pooled equimolarly and sequenced on an Illumina MiSeq using the MiSeq reagent kit v2 (500 cycles). Extensive details on the experimental setup, sampling process, and lab work and supplemental material are provided at https://git.io/JD82n. The number of reads varied from 51,156 to 150,856 across the 120 samples, with GC contents ranging from 54.8% to 57.4% and a length of 251 bp prior to trimming. The raw sequencing data were analyzed in QIIME2 (7) v2021.8 using the DADA2 pipeline (8) as described at https://tinyurl.com/4w8xvya5, with default settings and the SILVA-132-99 (9, 10) database for taxonomic assignment. Figure 1 shows relative abundances of the main phyla as identified by 16S amplicon sequencing.

The analyses showed that the bacterial and archaeal community compositions were significantly affected by cultivar for both rhizosphere and rhizoplane (*P* = 0.001). The generated amplicon data sets (3 rhizocompartments of 4 wheat cultivars) can be explored in future studies, potentially revealing impacts of different cultivars on the wheat soil microbiome and elucidating patterns associated with better plant growth performance.

### Data availability.

The 16S rRNA gene amplicon data set has been deposited at GenBank and can be accessed in the SRA under the BioProject accession number PRJNA806868.
